# Qualitative Evaluation of a Text Messaging Intervention to Support Patients With Active Tuberculosis: Implementation Considerations

**DOI:** 10.2196/mhealth.3971

**Published:** 2015-02-27

**Authors:** Sarah J Iribarren, Katherine A Sward, Susan L Beck, Patricia F Pearce, Diana Thurston, Cristina Chirico

**Affiliations:** ^1^School of NursingColumbia UniversityNew York, NYUnited States; ^2^College of NursingUniversity of UtahSalt Lake City, UTUnited States; ^3^School of NursingLoyola UniversityNew Orleans, LAUnited States; ^4^Salt Lake County Health DepartmentSalt Lake City, UTUnited States; ^5^Region V Tuberculosis Control ProgramVicente LópezArgentina

**Keywords:** mHealth, tuberculosis, sociotechnical evaluation, text messaging

## Abstract

**Background:**

Tuberculosis (TB) remains a major global public health problem and mobile health (mHealth) interventions have been identified as a modality to improve TB outcomes. TextTB, an interactive text-based intervention to promote adherence with TB medication, was pilot-tested in Argentina with results supporting the implementation of trials at a larger scale.

**Objective:**

The objective of this research was to understand issues encountered during pilot-testing in order to inform future implementation in a larger-scale trial.

**Methods:**

A descriptive, observational qualitative design guided by a sociotechnical framework was used. The setting was a clinic within a public pulmonary-specialized hospital in Argentina. Data were collected through workflow observation over 115 days, text messages (n=2286), review of the study log, and stakeholder input. Emerging issues were categorized as organizational, human, technical, or sociotechnical considerations.

**Results:**

Issues related to the intervention included workflow issues (eg, human, training, security), technical challenges (eg, data errors, platform shortcomings), and message delivery issues (eg, unintentional sending of multiple messages, auto-confirmation problems). System/contextual issues included variable mobile network coverage, electrical and Internet outages, and medication shortages.

**Conclusions:**

Intervention challenges were largely manageable during pilot-testing, but need to be addressed systematically before proceeding with a larger-scale trial. Potential solutions are outlined. Findings may help others considering implementing an mHealth intervention to anticipate and mitigate certain challenges. Although some of the issues may be context dependent, other issues such as electrical/Internet outages and limited resources are not unique issues to our setting. Release of new software versions did not result in solutions for certain issues, as specific features used were removed. Therefore, other software options will need to be considered before expanding into a larger-scale endeavor. Improved automation of some features will be necessary, however, a goal will be to retain the intervention capability to be interactive, user friendly, and patient focused. Continued collaboration with stakeholders will be required to conduct further research and to understand how such an mHealth intervention can be effectively integrated into larger health systems.

## Introduction

Adherence to long-term medication therapy in the outpatient setting remains a global health challenge, particularly for tuberculosis (TB) treatment [1,2]. The World Health Organization (WHO) and others have called for patient-centered approaches that tailor interventions to meet patient needs [3-6]. Regular supervision and support provides opportunities for education and problem monitoring, and promotes medication adherence [6]. Directly observed therapy (DOT) has been the predominant TB medication management strategy since the 1950s [1,2]. However, DOT is challenging for patients and health care providers, due to limited resources, operation expenses, and daily travel burden [7-9].

Mobile health (mHealth) interventions utilize portable devices, such as mobile phones, to provide health services [10,11]. Mobile phones are increasingly prevalent across the globe [12] and there are a growing number of researchers assessing mHealth’s impact on health outcomes [13,14]. Promising evidence suggests that mHealth interventions can enhance health services in low- and middle-income countries [15-17]. However, uptake at a larger scale can be slow despite growing evidence of the potential benefits [18,19]. Previous studies of mHealth interventions in TB management have focused on DOT using the mobile phone’s video features [20-22], traditional phone calls to mobile phones to remind patients to take their medication [23], or sending short message service (SMS) text messages asking participants to respond with the time they took their medication [24]. The most common type of mHealth intervention reported in the literature is one-way SMS text messaging [10].

Although mHealth technology offers promise to aid in management of chronic conditions, there remains insufficient evidence to inform larger-scale implementation [25]. Leading experts recommend rigorous research of mHealth potential, as well as the implementation challenges, and warn against skipping outcome evaluations, which could threaten the understanding of the long-term value of mHealth [26]. Small-scale and pilot implementations are needed to provide evidence of acceptability and feasibility and can suggest ways to improve mHealth interventions to avoid larger-scale implementation pitfalls [25,27].

The objective of this research was to understand implementation issues encountered during pilot-testing, and to identify system improvements that will inform future implementation in a larger-scale trial.

## Methods

### Study Design

The research was based on descriptive observational qualitative design [28,29] guided by an adapted sociotechnical framework (see below). This study was the second phase of an interventional study that explored feasibility, acceptability, and initial efficacy of the interactive TextTB intervention to support patients with active TB (described below) [30]. Data were collected through workflow observations over 115 days and were tracked in a study log (eg, process, problems, barriers, and one-on-one and team meeting discussions). Text messages (n=2286) were reviewed for content related to technical challenges, and verbal and written stakeholder input—feedback to questions based on the sociotechnical framework—was collected. The setting was a clinic within a public pulmonary-specialized hospital in Buenos Aires, Argentina. The study was approved by the University of Utah Institutional Review Board (IRB) and an independent research ethics board of Hospital Italiano, in Buenos Aires, Argentina.

### Theoretical Framework

For this study, sociotechnical models from Cornford et al [31] and Barber et al [32] were adapted. These models integrate classic Donabedian Structure-Process-Outcome quality improvement concepts with a sociotechnical perspective to understand health information technology implementation outcomes [31,32]. In addition, a Rapid Assessment Process (RAP) was used as a process guide [33]. The RAP, adapted for informatics evaluation from ethnography and other qualitative methods, has been shown to be useful for explaining health technology implementation success or failure, and for providing feedback for system improvements [33]. Informatics interventions, including mHealth interventions, often occur in naturalistic settings where certain variables are outside of the investigator's control. The basic definitions, matrix structure, and the framework from the models were adapted a priori to articulate variables relevant to this specific mHealth intervention (see Table 1).

**Table 1:** Theoretical framework based on the sociotechnical approach^a^.

	Technical approach	Social approach	
	System function	Human perspective	Organizational context
Structure	Technical detail and content: computer-based intervention details Hardware and software setup User requirements for the intervention	Adapting work conditions/requirements of intervention implementation Skill level or training needed (eg, computer skills) Work conditions and staff patterns	Requirements for sustainability: costs, management, and equipment needs Cost of the intervention Organizational/technical support for management and equipment Management (eg, team required)
Process	Information processing What system can capture How organized Correct and valid Functions of software	Participation of patient/health care team, social interaction Team’s participation in tasks Process of sending, receiving, responding to text messages Shift in attitudes/beliefs	Altered practice and delivery of service Workflow changes and monitoring Aspects of intervention (ie, how it fits) Protocol: process/steps when patient not responding Communication interactions
Outcome	Technical performance: efficiency and reliability Hardware/software issues Reliability of system Ability to send, receive, store, and retrieve data Intervention appropriateness	Quality of service and individual outcomes Perceptions of quality (patients, staff) Outcomes for individuals (eg, adoption by staff) Changes in workflow, workload Text message relevance	Global effect: lessons learned, potential application to other settings Lessons learned Implementation process Balance tech/human perspective Steps needed to implement larger trial

^a^Adapted from Cornford et al [31] and Barber et al [32].

### The Intervention and Team

The TextTB intervention pilot study occurred from December 2011 through April 2013. Details of that study are reported elsewhere [30,34]. In brief, participants were randomized to the intervention group (TextTB, n=18) or control group (paper documentation, n=19) for the first 2 months of active TB treatment. Participants in the TextTB group were asked to (1) send an initial SMS text message to confirm connection with the system at enrollment, (2) text daily to confirm they took their TB medication that day, and (3) text any questions or concerns. They received confirmation that their text messages were received, or they received query messages if they failed to send notifications. They also received twice weekly educational text messages that were based on the Information-Motivation-Behavior skills model [34-36]. The team members were the study principal investigator (PI), a regional TB director/pulmonologist, the lead regional TB social worker, a regional TB staff member, two TB-specialized clinic registered nurses (RNs), and the hospital TB program director/pulmonologist.

### Technical Platform

FrontlineSMS version 1.6.16.3 was the platform selected to send, receive, and manage text messages [37]. FrontlineSMS is an open-source, free software program that is installed on a laptop and functions with a GSM modem and a local SIM card. The GSM modem fits into the laptop and holds the SIM card. The SIM card includes the imbedded chip required for cell phone transmission [38]. The card contains identification numbers, controls which phone services the user can access, and can be moved between mobile devices. Together, the GSM modem and SIM card allow the computer to function like a mobile phone to send and receive text messages across a mobile phone network [38].

### Implementation

Hands-on training and written directions in Spanish on how to operate the FrontlineSMS platform were provided by the PI to four team members. One team member primarily managed the daily patient interactions, while another did so on occasion or during vacation periods. Two pulmonologists were available for consultation for participant questions that were technical or needed expert advice (eg, recommendations for potential allergic reaction). Other nonparticipating hospital staff members were introduced to the intervention during a hospital-wide conference focusing on TB case presentations and current and future goals for TB management.

### Analysis

Data from the combined sources (eg, study log, text messages, and stakeholder feedback) were assessed for implementation issues and categorized based on the evaluation framework (see Table 1). An iterative, interpretive, and flexible process was applied as recommended for RAP methods [33]. Analytic validity was strengthened with *member checking* through local stakeholder review, group consensus, and by drawing from multiple data sources. The PI conducted the initial categorization based on theoretical framework definitions. The other investigators reviewed the categorizations, which were iteratively refined until group consensus was achieved. An onsite champion (eg, local expert) who could serve as a liaison to clinical staff facilitated the evaluation process and provided member checking. Ash et al noted the importance of an onsite expert as well [33]. Based on the specific issues identified in this study and the pragmatic experience of the investigators, potential modifications for a larger-scale trial implementation were outlined. Analysis was supported using ATLAS.ti version 6 (GmbH, Berlin, 2009).

## Results

### Organizational Considerations

#### Overview

Organizational considerations included issues that could impact sustainability, delivery of services, and potential application to other settings. Issues encountered that impacted the intervention included equipment security, intervention-related costs, and workflow changes.

#### Equipment Security

The original plan was for the study laptop to be located at the nursing station, but it became apparent that the laptop might be unattended at times throughout the day, raising the risk for a security breach or outright theft. Therefore, the laptop was moved to the regional TB office located upstairs in the same building. Security concerns also required that the laptop be turned off and put away in a locked cabinet when study staff members were not present, which meant that the software was available only during clinic office hours, rather than around the clock. Participants were informed that responses would be provided only within clinic hours, Monday through Friday, and emergencies were to be directed through standard routes. However, powering down the program caused other problems—see technical and sociotechnical consideration sections.

#### Financial Considerations

The study had associated set-up costs. The FrontlineSMS software was open source and available free of charge [37]. However, there were costs associated with the purchase of the laptop computer, GSM modem, and SIM card. The GSM modem was replaced due to technical issues, incurring additional cost.

The majority of ongoing costs were associated with text messaging over a mobile phone network (ie, SMS text messaging). A majority of participants reported having basic feature mobile phones (26/37, 70%) and pay-as-you-go mobile phone plans (22/37, 59%). In Argentina, text messages are free to receive and ranged from ARS (Argentinian peso) $0.60 to $1 (US $0.10-$0.23) to send for pay-as-you-go service plans, according to the participants and mobile phone service provider websites. However, these fees could vary substantially due to service provider promotions or by purchasing a package with unlimited text messaging for a given number of days. Based on cost range per text message, the intervention averaged US $12.80 to $29.44 per participant for a 2-month period for messages sent and received.

Although there are options for online mobile phone service providers to send text messages at a discounted rate, there were logistical problems with this service. Initially, credit was purchased as a bundle from one of the suggested online mobile phone credit providers (Clickatell). However, in order to use the credit in Argentina, the phone number had to remain a US or European number, which would have required the patients to send messages to an international number, greatly increasing the costs and potentially challenging the legitimacy of the intervention being conducted by local team members. Therefore, we chose to use a local phone number. Other cost-related considerations included two phones reported as lost or stolen, and credit used up sooner than anticipated in some instances, in which case the monthly credit was added to the patient account early.

#### Workflow Changes and Monitoring

Initially, the intervention was to be managed by the nurses. However, because the laptop had to be moved to a more secure area upstairs, the nurses were unable to consistently leave their area to run the intervention. The technician volunteered to be the primary operator of the messaging platform. The regional director managed the platform when others were on sick leave or on vacation. An Excel file was developed to help visually track participant stage of treatment and notifications, and to clearly identify those who did not send notifications. The technician estimated between 15 minutes to 1 hour per day was required to review and respond to the text messages. This time was broken up into two to three intervals between work responsibilities.

Another workflow impact resulted from tracking and managing mobile phone credit compensation. Participants were provided with mobile phone credit at study onset and at the beginning of the month to compensate for cost of texting daily and at the end of each month for study participation. Although adding credit was easy, it required leaving the hospital to go to a kiosk nearby where credit could be added with the phone number and name of the mobile provider. This task was additionally complicated because participants were enrolled in the study at various times and cash was required for the transaction.

Although not directly related to the mHealth intervention, the paper-based medical charting system in place was a challenge for collecting final treatment outcomes. Manual chart review, to identify and collect treatment outcomes, was time consuming and accompanied by occasional missing or incomplete data. When considering future trials at a larger scale, the integration of the mHealth data into the paper-based medical records will be challenging or impossible.

#### Medication Shortage

There was an unexpected TB medication shortage at the regional and national TB program levels during the study. The shortage and outage of some medication was documented in the study log from December 1, 2012 to the last text message regarding the medication shortage sent on March 20, 2013. However, availability or amount of stocked medication may have been an issue for a longer period. Because of the shortage, medication was distributed to participants for shorter time periods, for example, 5 days or 2 weeks rather than the standard 1- to 2-month supply. In some instances, only one of the medications was allocated and patients were told to keep checking back for the missing medication. Although not the focus of our study, the medication shortage may have impacted treatment outcomes, and text messages were added to inform participants when medication became available.

### Human Perspective Considerations

#### Overview

Human/social considerations focused on requirements for implementation (eg, training), team participation, change in attitudes or beliefs, and perception of the quality of the intervention.

#### Training

It was necessary to train study personnel to use the messaging platform, and an extended period of technical support was required. Training was provided by the PI to the regional TB director/physician, a social worker, two nurses, and a technician. One nurse and the technician were computer novices and required instruction on basic computer functions (eg, how to save a file, how to copy and paste text). All trained team members were able to review and send text messages using the platform. All intervention participants knew how to send text messages. Participants were given verbal and written instructions on the desired format for notification messages. However, there were still problems with inconsistent formatting of notification messages (discussed below in the auto-confirmation section).

#### Team Participation and Process

Responding to patients’ questions was often a collaborative process. The TB technician solicited assistance from team members when necessary. The technician had worked in the hospital for nearly 20 years, had conducted TB testing, had basic TB knowledge, and was familiar with the hospital/clinic setting. The office was without walls or dividers and the regional TB director (pulmonologist/TB specialist) and TB social worker were seated nearby and could provide rapid assistance.

#### Attitudes and Beliefs

The team indicated that the ideal would be for patients to be referred to local centers for close monitoring during their TB treatment course. However, they all agreed that the intervention appeared to be beneficial for those receiving treatment by self-administration and especially for those who lived in rural or semirural settings where access to health care was challenging. The technician, who primarily managed the intervention, indicated that he felt he was truly able to help participants through this mode of delivery. He noted that the intervention seemed to be very useful to participants, especially for those who had many questions, concerns, or needed advice.

Some of the staff, however, believed that self-administration functioned well despite a documented high rate of treatment abandonment. As a result, there were some staff who hesitated to act with respect to potential abandonment before a full month had passed. In one case, the patient contact information was not entered into the medical record and, therefore, follow-up could not be initiated. The intervention did serve to identify a patient who had been documented in the notification records as abandoning treatment, but who had actually transferred to a different health care facility and had noted doing so in one of her text messages.

### Technical System Function (Platform) Considerations

#### Overview

A number of the technical issues encountered during the pilot test were able to be remedied, while others will need further consideration for a larger-scale trial. The technical issues, including software quirks, errors in data exporting, and software inefficiencies affected the intervention flow and analysis of results, as well as the reliability of the program.

#### Organizing Messages

The platform software allowed messages to be organized in multiple ways (eg, by contact, sent or received, or all). However, it was identified that a “0” needed to be added to the beginning of each mobile phone number in order to match the messages to an individual. In addition, although two local area codes were used interchangeably for calls within the province, the software recognized only one (eg, 11, and not 15).

#### Scheduling of Reminders

**Figure 1:**
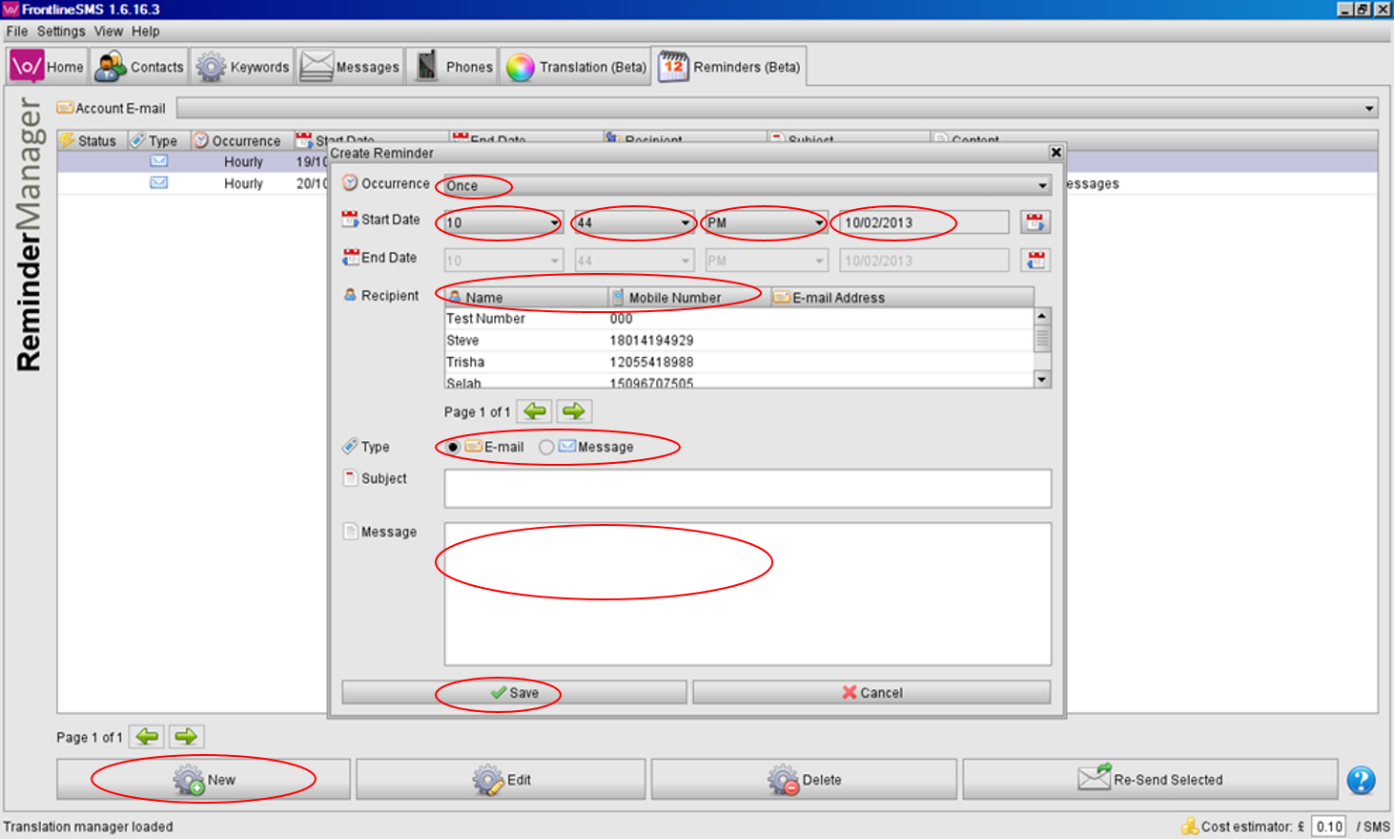
Steps required to input and create an automatic educational text message.

The reminders software feature was used to set up the package of educational messages for automatic delivery at predetermined times, twice weekly. Figure 1 illustrates the steps necessary to create one message (eg, select date, frequency, add content). Patients entered the study on a rolling basis, therefore, the package of messages had to be created for each participant. It took 10 to 15 minutes to create each package. By trial and error it was identified that the software had a maximum capacity of 100 reminders. The system did not provide a notification or alert to indicate why new messages could not be created or that maximum capacity was near or reached. Once this issue was identified, sent messages had to be deleted at regular intervals for new ones to be added.

#### Impacting Cost and Data Analysis

**Figure 2:**
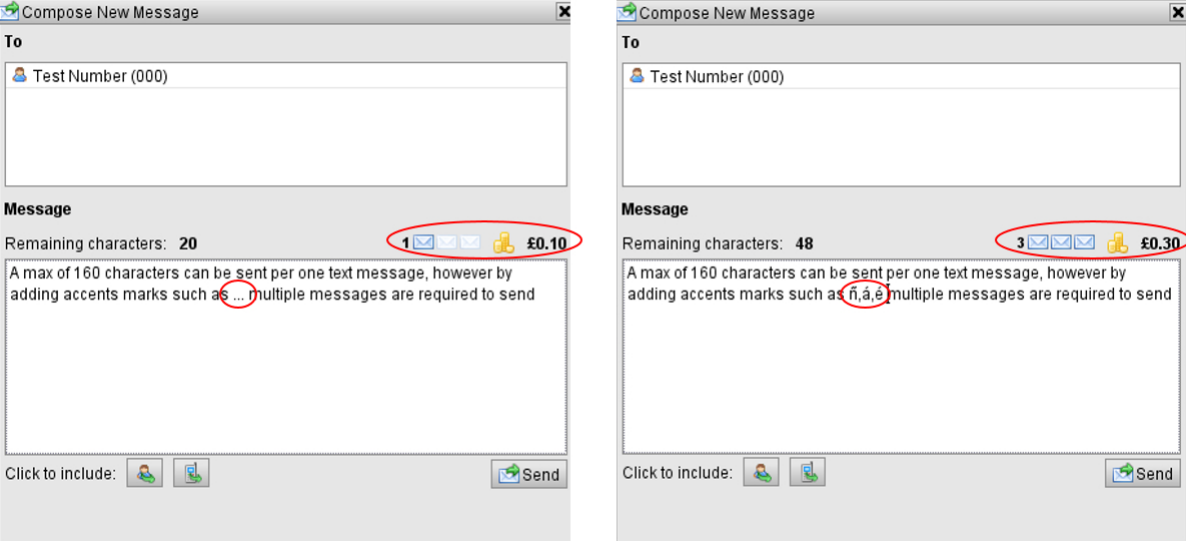
Number of texts and associated costs without (left) and with (right) accent marks.

Accents marks, commonly used in the Spanish language, were recognized as multiple characters. Despite not exceeding the 160-character limit, this miscount cued the platform to send multiple messages (see Figure 2). Once this problem was identified, accent marks were removed from the educational messages.

In addition, problems were encountered when exporting messages with accent marks for analysis. The software has a function to export data into an Excel file. However, letters with associated accent marks were changed to unrecognizable characters during this process. One example is the Spanish word for “I took”, which is “Tomé” that was changed to “TomÃ©.” The messages had to be reviewed individually and corrected in the exported data file, resulting in increased time for data analysis.

In addition, messages were time stamped 2 hours earlier than when they were sent or received. Consultation with the program staff suggested that the cause of the time discrepancy might have been because the study computer was set up in a different time zone. Changing the time zone on the computer did not correct the problem. This issue warrants further investigation to assure precision time stamping in the future.

#### Reliability of Software

Inconsistencies were identified between the confirmation of messages sent within the platform and messages delivered after messages were not received on the study mobile phone. It was determined that when the mobile phone credit was depleted, messages were still shown as sent within the platform even though they were not sent by the mobile phone carrier. There was no link between the mobile phone carrier or credit amount remaining on the SIM card and the FrontlineSMS software. To address this problem, the mobile credit balance had to be regularly checked on the service provider website. This was another task to remember to do on a regular basis to avoid episodes where messages were believed to be delivered, but were not.

#### Larger System Issues Impacting Technical System Function

Network coverage variability and Internet and electricity outages were outside of the investigators’ control, but impacted intervention implementation. The impact of data loss due to poor or no network coverage was minimal for the pilot study, but could be significant for a larger-scale implementation. There were participants who reported loss of network coverage when traveling as the cause for missing days’ worth of notifications. In addition, one of the main mobile phone service providers had a 3-day outage and no messages could be sent or received, which likely resulted in loss of data. The company provided a compensation of ARS $10 (about US $3.5) for the loss in service.

In addition, there were 10 days logged in the field notes during which there was no Internet access and days without electricity. Access to the Internet is required to download the FrontlineSMS program, but after installment, Internet access was no longer required because the software sends messages over a mobile phone network instead of over the Internet. Disruption in Internet access could be a concern for cloud-based programs. For several days, electricity was out for all, or part, of the work day. The loss of electricity did not cause a delay in messages being received or sent because the laptop had a long battery life. However, extended periods without electricity could be a significant problem for larger-scale implementations. In addition, when the radio was on in the office, a notable static disturbance in the music was heard when texts were received.

### Sociotechnical Considerations

#### Overview

Sociotechnical considerations reflected the interactions between people and technology and focused primarily on issues that affected study participants. Issues included inconsistent message delivery, deletion of reminder settings, challenges to using keywords for auto-confirmation messages, and messages delivered out of sync.

#### Inconsistent Message Delivery

For the first 2 months, reminder messages were sent to participants with some replying that they had sent their notification earlier. It was eventually identified that depending on the order of opening the software (eg, modem first and then FrontlineSMS, or vice versa) messages would go to the modem and not be delivered to the messaging platform. It was later recognized that the modem had a storage maximum at which time messages needed to be deleted in order to receive new ones. Initially, the modem inbox was reviewed daily and messages (n=55) were manually transferred into the database. Then a new modem (different model) was incorporated into the trial and there was fewer instances of data not being received. Remaining issues appeared to be caused by problems with service coverage, rather than the modem.

#### Deletion of Reminder Settings

The educational messages set up using the reminder feature were also not consistently delivered at the prespecified time and date. When reminders were sent, a green check mark was displayed to the left of the message row in the software window. The reminders that were not checked had to be reviewed and resent. It was later discovered that powering down the program caused the automatic reminder settings to be erased. As previously mentioned, the study computer had to be powered off and stored because of security issues.

#### Challenges to Using Keywords to Send Auto-Confirmation Messages

The keyword feature was used to set up auto-reply messages to daily notifications of self-administration of medication. In order for the software to recognize a keyword, it must be the first word of the message. It was identified that the software was also sensitive to case, accent marks, and punctuation. As a result, multiple similar keywords were added (eg, upper-/lowercase, with/without accent marks) in response to the different ways participants formatted messages. Although participants received both verbal and written instructions for the formatting of notification messages (eg, “Tome 3R 4B” for “I took 3 red and 4 white pills”), message format often varied (eg, “hello”, “good day”, or other first words). This led to failure of the system to auto-confirm receipt of notification, requiring the technician to manually review messages and respond. Some keywords were added in attempt to accommodate communication patterns (eg, “hola!!!”). However, the number and variety of keywords also led to auto-confirmation messages being sent in error (n=17) when, for example, a question rather than a notification was received.

#### Messages Delivered Out of Sync

Another outcome to shutting down the study computer nightly and over the weekend due to security reasons was that some messages were delivered out of sync. That is, if a message was sent outside of the office hours, the auto-response was delayed until the software was powered on. For example, if a participant sent notifications over the weekend, multiple acknowledgment messages were sent first thing Monday morning. These messages that were delivered out of sync caused confusion for some participants. Some participants texted back that they received a confirmation message, but had not yet taken their medications that day.

##  Discussion

### Principal Findings

The goal of this research was to identify and describe practical issues that need to be considered before widely disseminating the TextTB intervention. This research uncovered generalizable issues that others may want to consider prior to developing and implementing an interactive mHealth intervention. Understanding what worked and what likely would not work at a larger scale is important for planning. Potential solutions were identified for some of the problems (see Table 2). The potential solutions need to be evaluated for effectiveness in future research.

**Table 2:** Identified implementation issues and potential solutions.

Issue	Potential solutions	
Adding phone credit was time consuming and credit was depleted early for some participants	Free texting	
		Contract with local mobile phone carriers Trial mobile messaging app that allows exchange of messages without SMS charge
Messaging platform shortcomings	Modify open-source software or consider other available platforms	
	Improve features	
		Message package upload and tailored delivery Alerts to health care team Visualization of treatment course
	Retain interactive feature to promote patient/health care personnel relationship	
	Identify or develop software that provides message selection options (eg, an app)	
		Structured messages (improve ease of patient reporting, maximize automaticity of program features, and reduce manual intervention) Free text option for questions and responses
Patient tracking	Further partner with stakeholders (eg, National TB Program director, local health care centers)	
	Have dedicated intervention management staff	
Sustainability	Economic evaluation	
	Confirm transfers to another health care facility	
	Case contact tracing	
	Involve health care provider in care management	
	Implement intervention for full treatment course	

### Intervention Strengths

The texting platform software allowed for a large number of contacts and enabled multiple view options. The software allowed interactivity, personalization, and messaging functionality without requiring access to the Internet. Although we attempted to make the intervention as automated as possible, the team also wanted to assure that it could allow personalized communication with the patients, and not be viewed as too automated.

### Intervention Areas to Strengthen

We experienced issues of delayed response and multiple acknowledgment messages. After this study concluded, we learned from other researchers (personal communication) that powering off the software can cause the reminder feature to be inactivated, which would explain the inconsistencies we experienced. Our solution was a manual review of sent messages and resending unsent messages. The solution for the other researchers was to leave the computers running continuously, although they have experienced issues caused by software updates or the computer accidentally being unplugged. However, in our situation the computer needed to be powered off and stored daily because of security issues.

Patients sent notifications that they took their medication in a number of ways, although verbal and written directions were provided, which caused inefficiencies and messages sent in error. For widespread dissemination, software that provides message selection options (ie, an app instead of basic messaging) might be useful. Selecting from structured messages, with an option to include free text questions, could improve patient reporting, maximize automaticity of program features, and reduce the need for manual responses. However, the use of structured messages may also make the intervention less personal.

The software was not built specifically for the application for which it was used in this study and, therefore, we expected to find some misalignment of features. A new Web-based version of FrontlineSMS, version 2, is now available. It is advertised as more intuitive, easier to use for creating and managing messages, and capable of managing larger volumes of messages [37]. However, in the new version, a number of key features we used, such as the translation manager and reminders, are currently not available. This exemplifies a challenge to implementing open-source software. It is difficult to purposefully use a previous version of software once the new one is released, and the new version may be quite different.

Even though sufficient credit for the intervention was added to patients’ mobile phones at the beginning of each month, running out of credit early was a problem for some participants. Texting and mobile phone use habits are unique to each individual. For example, one participant indicated that when she had available funds, her usual mobile phone habit was to purchase a package that allowed unlimited calls and texting for 5 days. She would use her phone as much as possible during these periods. She reported having to use her mother’s phone during the study because she had used all her credit within a short time. In addition, the workflow associated with adding phone credit for each patient at local kiosks, although feasible for a pilot study, would likely be prohibitive on a larger scale. An ideal solution for this issue would be to establish a free to-text-in number.

Easy access to the computer is an essential feature for workflow integration. The original plan was for the intervention to be managed by the nurses. When equipment security concerns required the laptop to be moved upstairs, the nurses were unable to consistently leave their area to run the program. Nurses are often overburdened with multiple tasks and patient interruptions. Leaving the nursing area daily to monitor the messages was not feasible. With large numbers of patients, the ideal solution would be to hire one individual with TB specialty knowledge to manage the messaging.

### Factors Unrelated to the Intervention

Potentially, extended periods of Internet and electrical outages could have caused major problems with the research. Internet and electrical outages can occur more often in low- and middle-income countries. Hoffman et al [21] reported challenges of Internet access in Kenya with downtime due to frayed network cables and slow system access. Because the software we used did not require the Internet to operate, outages experienced during the study period were not a limiting factor to the continuity of intervention delivery. We used a computer that had a long battery life. There were a few participants, however, that reported depleted mobile phone batteries after long periods of power outages. Extended electrical outages could certainly be anticipated to cause problems for any mHealth intervention.

Loss of data was a major frustration. Data loss is not unique to the technology used in this study. Hoffman et al [21] reported technical and transmission problems resulting in an estimated 25% loss of data, and Wade et al [22] reported technical problems with variable phone signal strength.

### Use of the Sociotechnical Framework

For this study, we adapted a framework [31,32] in which sociotechnical concepts (ie, technology, person, organization) were matched to classic structure-process-outcome concepts to create a matrix. There were limitations to our approach. The matrix was simple and explicit, but the interaction between components, which is a key part of sociotechnical systems theory, remained implicit and could have been overlooked. The matrix also did not account for broader contextual issues like electrical outages or a national medication shortage, and the approach does not explicate interpersonal interactions. Sociotechnical approaches have provided a powerful framework within which to analyze reasons for uptake and performance with many types of information and communications technology, and can contribute to the design of those systems [39]. Much of the richness and nuance of the sociotechnical approach was lost with our approach. However, it was pragmatically useful for the purposes of analyzing and categorizing implementation issues.

The field of mHealth is rapidly evolving, and frameworks designed specifically to evaluate mHealth interventions are beginning to emerge. For example, Leon et al [40] applied a health system framework to assess community-based health services system challenges in South Africa. Their framework included larger context and sustainability issues and identified four key dimensions: government stewardship, organizational systems, technical systems, and financial systems. Hirschhorn et al [41] and Aranda-Jan et al [42] conducted systematic reviews to identify potentially important domains for scaling up and evidence of which mHealth components worked and did not work in Africa. Mohr et al [43] focused on principles from behavior change theories in the context of mHealth.

### Limitations

The purpose of this research was to identify key areas to improve for further testing of the intervention in a larger sample. We succeeded in identifying and developing solutions for most of the problems we encountered, although a few issues remained unresolved. Issue tracking, determining the cause of problems, and developing solutions occurred on an ad hoc basis. Although informatics experts were a part of the PI dissertation committee, on-the-ground IT support was limited. As such, some of the issues encountered may have been managed proactively with a local IT expert. In addition, the cost estimate was for the texting component only, and did not factor in staff time—such estimates are recommended for the next research phase.

### Conclusions

Intervention challenges were largely manageable during the pilot study, but when evaluating these challenges for a larger-scale trial, issues to be addressed were identified with potential solutions outlined here. These findings may help others considering implementing an mHealth intervention to mitigate these challenges. Although some of the issues may be context dependent, other issues such as electricity and Internet outages, limited supplies, and human resources are not unique issues to our setting. Release of new software versions does not necessarily result in solutions for certain issues, particularly when specific features are removed. As such, other software options will be considered prior to moving to a larger-scale study. Improved automation of some features was recognized as necessary for use with a larger sample, however, a goal will be to retain the intervention capability to be interactive, user friendly, and meet the needs of the patients and the health care team. Continued collaboration with stakeholders will be required to conduct further research and to understand how such an mHealth intervention can be effectively integrated into larger health systems.

## Abbreviations

DOT: directly observed therapy
IRB: Institutional Review Board
mHealth: mobile health
NIH: National Institutes of Health
NINR: National Institute of Nursing Research
NRSA: National Research Service Award
PI: principal investigator
RAP: Rapid Assessment Process
RN: registered nurse
SMS: short message service
TB: tuberculosis
WHO: World Health Organization
